# KDELR2 is necessary for chronic obstructive pulmonary disease airway Mucin5AC hypersecretion via an IRE1α/XBP‐1s‐dependent mechanism

**DOI:** 10.1111/jcmm.70125

**Published:** 2024-10-04

**Authors:** Xiaojuan Wu, Fawang Du, Aijie Zhang, Guoyue Zhang, Rui Xu, Xianzhi Du

**Affiliations:** ^1^ Department of Respiratory and Critical Care Medicine The Second Affiliated Hospital of Chongqing Medical University Chongqing China; ^2^ Department of Respiratory and Critical Care Medicine Suining Central Hospital Suining Sichuan China; ^3^ Basic Laboratory, Key Laboratory of Metabolic Diseases Suining Central Hospital Suining China

**Keywords:** COPD, endoplasmic reticulum, KDELR2, Mucin5AC, UPR

## Abstract

Airway mucus hypersecretion, a crucial pathological feature of chronic obstructive pulmonary disease (COPD), contributes to the initiation, progression, and exacerbation of this disease. As a macromolecular mucin, the secretory behaviour of Mucin5AC (MUC5AC) is highly dependent on a series of modifying and folding processes that occur in the endoplasmic reticulum (ER). In this study, we focused on the ER quality control protein KDEL receptor (KDELR) and demonstrated that KDELR2 and MUC5AC were colocalized in the airway epithelium of COPD patients and COPD model rats. In addition, knockdown of KDELR2 markedly reduced the expression of MUC5AC both in vivo and in vitro and knockdown of ATF6 further decreased the levels of KDELR2. Furthermore, pretreatment with 4μ8C, an IRE1α inhibitor, led to a partial reduction in the expression of KDELR2 and MUC5AC both in vivo and in vitro, which indicated the involvement of IRE1α/XBP‐1s in the upstream signalling cascade. Our study revealed that KDELR2 plays a crucial role in airway MUC5AC hypersecretion in COPD, which might be dependent on ATF6 and IRE1α/XBP‐1s upstream signalling.

## 1 INTRODUCTION

Chronic obstructive pulmonary disease (COPD) is considered a treatable and preventable disease characterized by persistent respiratory symptoms and progressive airflow limitations caused by exposure to noxious gases or particles. The World Health Organization (WHO) predicts that COPD will become the third leading cause of death by 2030.^1^ The progression of COPD leads to a drastic reduction in quality of life and worsens its economic and healthcare burden. Airway goblet cell hyperplasia and mucus hypersecretion are the distinguishing pathogenesis of COPD, which is defined by the limitation of airflow and pathologically by recurrent injury and inappropriate epithelial repair in airways.^2^ As one of the major pathologic features of COPD, excessive mucus synthesis is strongly associated with impaired lung function and exacerbation of COPD.^3^

Mucins are produced and secreted by goblet cells on the surface epithelium and by mucous cells in the submucosal gland. A previous study showed that goblet cells were more abundant in the airways of COPD patients, which can lead to elevated mucus hypersecretion.^4^, ^5^ Excessive mucus synthesis further results in airflow limitation and pulmonary ventilation dysfunction in COPD patients. Mucin5AC (MUC5AC), which is mainly synthesized and secreted by airway goblet cells, dominates the airway gel‐forming mucus. MUC5AC is a large multidomain oligomeric secretory molecule. The level of MUC5AC is markedly upregulated in the bronchiolar epithelium of patients with COPD,^6^ which is considered a hallmark of chronic lung diseases. Cigarette smoke and lipopolysaccharide (LPS) upregulates the expression of MUC5AC by leading to a prolonged inflammatory response.^7^ Acidification of the airway microenvironment caused by MUC5AC significantly reduces the bactericidal efficacy of commonly used antibiotics.^8^ This further results in colonization by the pathogen, persistent infection, and increased mucus secretion, which makes mucin hypersecretion an independent risk factor for COPD progression and mortality.^9^

The endoplasmic reticulum (ER) is an important subcellular organelle in the mammalian cells. The folding and secretion processes of the secretory proteins, polysaccharides, phospholipids and cholesterol are closely related to the normal capacity of the ER. Impairment of these processes by external cellular stimulation results in perturbed ER homeostasis, commonly known as ER stress. This stress can lead to an accumulation of unfolded or misfolded proteins within the ER lumen and trigger the activation of the unfolded protein response (UPR).^10^ Once ER stress is engaged, the UPR is subsequently triggered to either restore ER homeostasis (also called adaptive UPR) or evoke cell death in the case of intensive ER stress.^11^, ^12^ A certain degree of exogenous stimulation might induce an integration stress response (ISR) of the ER. This response restores proteostasis when unfolded proteins lead to the activation of ER transmembrane sensors: inositol‐requiring kinase 1 (IRE1), protein kinase RNA‐like endoplasmic reticulum kinase (PERK) and activating transcription factor 6 (ATF6), each of which binds together with the molecular chaperone glucose regulated protein 78 (GRP78). An in vivo study demonstrated that the ER stress sensor protein IRE1, which is localized in airway mucous cells, plays an important role in allergen‐induced mucin overexpression.^13^ In vitro experiments on human nasal epithelial cells also elucidated a possible relationship between mucin hypersecretion and ER stress.^14^

Luminal chaperones and ER‐resident proteins carry a C‐terminal Lys‐Asp‐Glu‐Leu (KDEL) sequence, which is important for the maintenance of proteostasis in the ER.^15^ KDEL receptors (KDELRs) contribute to the maintenance of ER homeostasis and quality control of the ER through the recovery of ER‐resident proteins and participate in the UPR.^15^, ^16^ Recent studies have demonstrated that mature goblet cells capable of producing sufficient amounts of mucus require a baseline level of the UPR with increased levels of chaperones and ER expansion.^17^, ^18^ However, in airway goblet cells, the specific mechanism of ER expansion induced by the baseline level of the UPR is still unclear.

Based on the evidence above, we propose that KDELRs might play a crucial role in airway MUC5AC hypersecretion, one of the key pathological manifestations of COPD, under the activation of a certain UPR. We designed in vivo and in vitro studies to reveal the potential mechanisms of KDELRs in airway MUC5AC hypersecretion.

## 2 MATERIALS AND METHODS

### 2.1 Bioinformatics tools

We used “COPD” as a keyword search for GSE datasets in the Gene Expression Omnibus (GEO) online database (https://www.ncbi.nlm.nih.gov/geo/), and GSE76925 was ultimately obtained, including comparative gene expression profiling between controls and COPD patients. GEOR2, an online tool of the Gene Expression Omnibus, was used to select differentially expressed genes (DEGs) from the gene expression data. DEGs were identified with the criteria of adjusted *p‐*value <0.05 compared with the control group, and upregulated DEGs were obtained. All the DEGs were used in further bioinformatics analysis.

### 2.2 Human sample acquisition

Lung specimens from patients with COPD and controls were obtained from the Suining Central Hospital between January 2022 and May 2023. Thirty‐seven patients who underwent segmental pulmonary resection to evaluate single pulmonary nodules that were highly suspected to be early‐stage lung cancer were included in our study. All participants were 35–80 years old. The patients were divided into a control group and a COPD group. The diagnosis of COPD was based on the Global Initiative for Chronic Obstructive Lung Disease (GOLD) criteria.^19^ The patients in the control group had no underlying chronic pulmonary diseases other than pulmonary nodules that were highly suspected to be early‐stage lung cancer. The exclusion criteria were as follows: (1) a history of α‐1‐antitrypsin deficiency or immunodeficiency disease and (2) any exacerbation and respiratory tract infection at least 1 month before admission. All lung tissues that were obtained were separated from the tumour by at least 3 cm and were confirmed to be normal lung tissues by a pathologist. All protocols were approved by the Ethics Committee of Suining Central Hospital, and written informed consent, including the study aims, was obtained from all individuals for publication.

### 2.3 Cell culture

Human mucoepidermoid carcinoma‐3 (Calu‐3) cells (ATCC, Manassas, VA) were grown in complete medium consisting of Dulbecco's modified Eagle's medium (DMEM, Gibco, NY, USA) supplemented with 10% fetal bovine serum (FBS, PAN, Germany) and 1% penicillin–streptomycin at 37°C in a 5% CO_2_ incubator. Human bronchial epithelial (BEAS‐2B) cells were cultured in DMEM/F12 (Gibco, NY, USA) supplemented with 10% FBS (PAN, Germany), penicillin (100 IU/mL), and streptomycin (100 IU/mL) at 37°C in a 5% CO_2_ incubator. Cells were passaged when 80%–90% confluent.

### 2.4 Animal model of COPD

Male Sprague–Dawley (SD) rats (300–350 g) aged 8–10 weeks were obtained from the Animal Experimental Center of Chongqing Medical University. The animal procedures were approved and performed according to the ethical standards of the Animal Ethics Committee of Chongqing Medical University. All of the rats were kept in an animal room maintained at 20°–24°C and fed standard animal food and water. Rats in the control group were in a room with normal temperature, similar to other rats and were given a tracheal instillation of sterile saline. Rats in the COPD group received intratracheal instillation of lipopolysaccharides (LPS, Sigma–Aldrich, USA) and were exposed to cigarette smoke for 4 weeks. Cigarette smoke exposure was given by passive smoking with 10 cigarettes (tar 9 mg, nicotine of flue gas 0.7 mg, carbon monoxide of flue gas 12 mg, Hongjinlong Filter tip cigarette, Hubei Tobacco Industry, China) 30 min a day for 5 days a week, and LPS (200 μg/100 μL) was instilled intratracheally on the first day, 14th day and the 29th day. Typical lung pathological changes were verified by haematoxylin and eosin stained pathological sections (Figure S1).

### 2.5 Bronchoalveolar lavage fluid collection

Rats were anaesthetised with 2% pentobarbital sodium (40 mg/kg) after the final exposure to cigarette smoke. Bronchoalveolar lavage fluid (BALF) in the left inferior lung was collected by lavage with 0.9% saline solution three times via the tracheal cannula, and 50%–60% of the lavage volume was recovered. Then, BALF samples were immediately centrifuged at 1200 rpm for 10 min at 4°C, and the supernatants were stored at −80°C for further analysis.

### 2.6 Adeno‐associated virus transduction in rats

To generate KDELR2 knockdown rats, male rats were transfected with 1.0 × 10^11^ viral copies of adeno‐associated virus (AAV) expressing short hairpin RNAs (shRNAs) targeting KDELR2 or negative control shRNA by intratracheal instillation under anaesthesia. AAV‐delivered shRNA and an AAV‐empty vector (AAV‐NC) were synthesized and constructed using the AAV6 Vector System (GeneChem, Shanghai, China), which was driven by the CMV promoter. The efficacy of KDELR2 downregulation in rat lungs was verified by RT–qPCR and Western blotting.

### 2.7 Pharmacological inhibitor administration

The IRE1α inhibitor 4μ8C (Selleck, USA) was dissolved in a mixture containing 5% DMSO. Some rats were administered 4μ8C (3.3 mg/kg) or DMSO (Sigma–Aldrich, USA) by daily intraperitoneal injection for 4 weeks.

### 2.8 Histological assessment

Lung tissues were fixed in 10% neutral‐buffered formalin for 24 h and embedded in paraffin. The sections (4 μm) of the paraffin blocks were stained with standard haematoxylin–eosin staining for analysis of bronchial inflammation and emphysema. Meanwhile, Alcian blue‐periodic acid‐Schiff (AB‐PAS) staining was performed on the tissues. Ten bronchioles with an internal diameter of 100–150 μm in each slide were found. PAS‐positive cells (goblet cells) and epithelial cells were counted under an optical microscope (Olympus, Japan). The results are expressed as the goblet cell percentage, which was calculated from the number of goblet cells in each bronchus divided by the total number of epithelial cells per bronchus. The following were the numerical scores for the quantity of PAS‐positive mucus‐containing cells in each airway: 0, <5% PAS‐positive cells; 1, 5%–25% PAS‐positive cells; 2, 25%–50% PAS‐positive cells; 3, 50%–75% PAS‐positive cells; and 4, >75% PAS‐positive cells. For each tissue slice, five fields of view were photographed.

### 2.9 Immunohistochemical analysis

The locations of MUC5AC, KDELR2 and KDELR3 expression were identified using immunohistochemistry. First, paraffin‐embedded sections were handled by submerging them in xylene and gradient alcohol solutions for deparaffinization and hydration after heating in an incubator at 60°C for 2 h and then sinking them in 10 mM citrate buffer for antigen recovery. Second, after endogenous peroxidase activity was quenched with 3% H_2_O_2_, sections were administered 10% goat serum blocking solution to minimize nonspecific binding of immunoglobulin. Finally, the sections were incubated with primary antibodies, anti‐MUC5AC antibody (1:500, Abcam, UK, ab3649), a rabbit polyclonal antibody against KDELR2 (1:100, Affinity Biosciences, China, Cat# DF4047) and a rabbit polyclonal antibody against KDELR3 (1:100, Affinity Biosciences, China, Cat# AF0758), at 4°C overnight. Subsequently, the slides were stained with a goat anti‐mouse/rabbit IgG secondary antibody‐HRP (1:500, Abcam, UK, ab97040, ab7090) at 37°C for 30 min and then incubated with a streptavidin‐peroxidase conjugate at 37°C for 30 min. The sections were stained with diaminobenzidine (DAB, ZSGB‐BIO, China, PV‐9000) and counterstained with haematoxylin after washing with phosphate buffer. ImageJ 1.8.0.112 software was used to evaluate protein expression. For each tissue slice, nine fields of view were photographed.

### 2.10 Cell viability assessment

A total of 1 × 10^4^ cells were seeded in each well of a 96‐well plate and cultured for 24 h. The cells were treated with different concentrations of neutrophil elastase (NE; Cusabio Biotech Co., China; CSB‐EP007587HU. 25, 50, 100 or 200 ng/mL) or 4μ8C (2 μM, 4 μM, 6 μM or 8 μM) when the cell confluency reached approximately 80%. At 6, 12, 24 and 48 h of treatment, the medium containing the NE or 48μC was discarded, and 90 μL of fresh medium supplemented with 10 μL of Cell Counting Kit‐8 (CCK‐8, ABclonal, China) solution detection reagent was added. Then, the samples were cultured at 37°C in an incubator for 1 h. The optical density was measured at 450 nm by an enzyme‐labeling instrument (Multiskan Sky 1530, Thermo Fisher Scientific, USA).

### 2.11 Enzyme‐linked immunosorbent assay

The BALF and cell culture supernatants were collected and used to determine MUC5AC concentrations via an Enzyme‐linked immunosorbent assay (ELISA) kit (Cusabio Biotech Co., Ltd., Wuhan, China; Abcam, UK, ab303761). The procedures were performed following the manufacturer's instructions. The absorbance was measured at 450 nm by a microplate spectrophotometer (Multiskan Sky 1530, Thermo Fisher Scientific, USA).

### 2.12 KDELR2 siRNA transfection in vitro

KDELR2 siRNA and negative control siRNA were obtained from Sangon Biotech Company (Shanghai, China). Cells were seeded in a 12‐well plate at a density of 1 × 10^5^ wells in medium containing 10% FBS and incubated overnight. Following serum‐free medium pretreatment, cells were transfected with KDELR2 siRNA or NC siRNA using Lipofectamine 2000 transfection reagent (Thermo Fisher Scientific, Waltham, MA, USA) according to the manufacturer's recommendations. The cells were then washed with phosphate‐buffered saline (PBS) 3 times after 6 h of transfection and incubated in complete culture medium for subsequent studies. RT–qPCR and Western blot analysis were performed to determine the efficacy of KDELR2 knockdown following KDELR2 siRNA transfection.

### 2.13 Immunofluorescence staining

After dehydration, dewaxing in xylene and rehydration in descending concentrations of ethanol: distilled water, the lung tissue sections were subjected to antigen retrieval in 10 mmol/L sodium citrate buffer and then heated for 20 min at 95°C. Slides with adherent cells were fixed in plates with 4% paraformaldehyde. Slides were washed in PBS 3 times and then permeated with 0.1% Triton‐X for 20 min. After blocking with 10% goat serum for 30 min at room temperature, lung sections and cells were incubated with primary antibodies against MUC5AC (1:500 dilution), KDELR2 (1:500 dilution), KDELR3 (1:500 dilution), or GRP78 (1:500 dilution, Abcam, UK, ab21685) overnight at 4°C. The sections and cells were washed with PBS 3 times incubated with the respective fluorescent secondary antibodies (Proteintech Company, China, Cat No: SA00003‐1; Cat No: SA00007‐2) at a 1:100 dilution for 1 h at room temperature and subsequently stained with DAPI (Beyotime, Shanghai, China, C1005) for 10 min in the dark. Images were visualized with a Dragonfly 200 confocal fluorescence microscope (Andor Technology, UK). For each tissue slice, nine fields of view were photographed.

### 2.14 Western blot analysis

Proteins from lung tissues and cells were extracted in RIPA lysis buffer supplemented with protease and phosphatase inhibitors (Beyotime, Shanghai, China) and quantified with a BCA protein assay kit (Beyotime, Shanghai, China). Equal amounts of protein from each sample were loaded and electrophoresed by SDS–PAGE and subsequently transferred to polyvinylidene fluoride membranes (Millipore, Merck, USA). The membranes were blocked in 5% BSA with Tris‐buffered saline (TBST) containing 0.1% Tween‐20 for 60 min, followed by overnight incubation at 4°C with primary antibodies (1:1000 dilution for anti‐IRE1α, anti‐pIRE1α, anti‐GRP78, a mouse monoclonal anti‐KDELR1 antibody (Sigma, Germany, SAB5200004), anti‐KDELR2, anti‐KDELR3, anti‐ATF6, anti‐XBP1, and anti‐XBP1s; 1:5000 dilution for anti‐β‐actin antibody; 1:10000 for anti‐GAPDH antibody). Then, the membranes were washed with TBST 3 times and incubated with horseradish peroxidase (HRP)‐conjugated goat anti‐mouse or anti‐rabbit secondary antibodies (1:2000 dilution, Proteintech Company, China) for 1 h at room temperature. Finally, all proteins were detected with enhanced luminol‐based chemiluminescence (ECL) reagents in a chemiluminescence detection system (Amersham Image 680, GE health, USA). Densitometry evaluation was performed using ImageJ 1.8.0.112 software.

### 2.15 Real‐time quantitative polymerase chain reaction (RT–qPCR) analysis

Total RNA was extracted from lung tissue or cells using TRIzol RNA Reagent (Ambion, Thermo Scientific, USA). We used PrimeScript RT master mix (ABclonal Biotechnology Co., China) to synthesize cDNA. The relative expression level of mRNA was then quantified using SYBR Green PCR master mix (ABclonal Biotechnology Co., China) by using an Applied BiosystemsTM I Cycler (Quantstudio 3, Thermo Fisher Scientific, USA). The sequences of the primers used for real‐time PCR were synthesized by Sangon Biotech. The qPCR primers for KDELR2 were 5′‐AGTGTGGAAGGAGGCTAGGAAAGTC‐3′ (forward) and 5′‐TCATAAGGCGTGGCATACAAGGC‐3′ (reverse); those for ATF6 were 5′‐ATTCAGTCTCGTCTCCTCGGTCAG‐3′ (forward) and 5′‐ATGGCATAAGCGTTGGTACTGTCTG‐3′ (reverse); and those for MUC5AC were 5′‐GGCAACATCAAGAAGAGCGGAGAG‐3′ (forward) and 5′‐TGTGGAGGTGGTACTGTCTGTCTG‐3′ (reverse).

### 2.16 Statistical analysis

The values are presented as the mean ± SD. All statistical analyses were performed with SPSS 21.0 software (SPSS Inc., USA) and GraphPad Prism 9.0.0.121 software (GraphPad Software Inc., San Diego, California, USA). Student's *t* test was used for comparisons between two groups. Comparisons among more than two groups were performed using one‐way analysis of variance (ANOVA). Statistical significance was defined as a *P* value <0.05.

## 3 RESULTS

### 3.1 KDELR2 and MUC5AC are upregulated and colocalized in the airway epithelial cells of COPD patients

**FIGURE 1:**
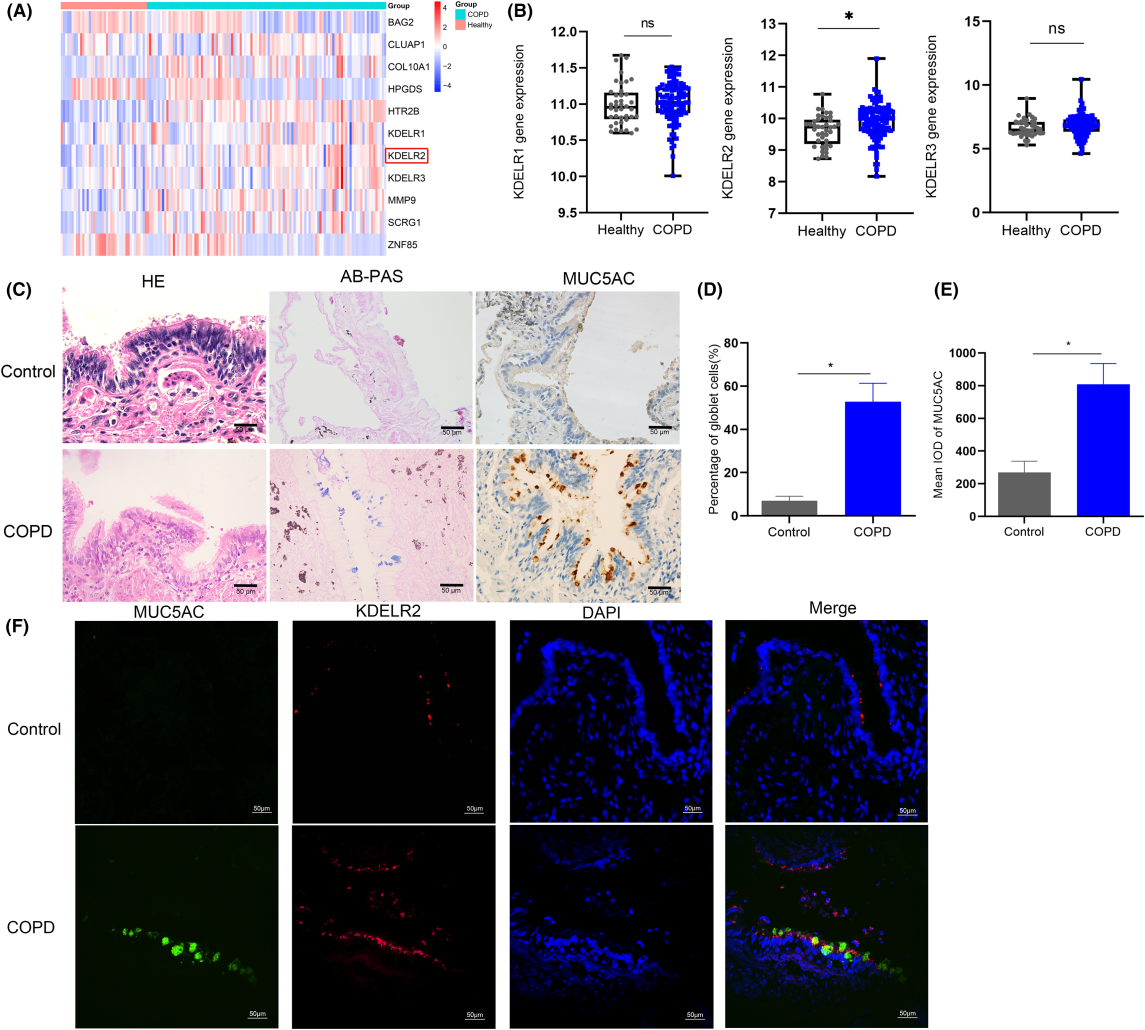
MUC5AC and KDELR2 expression levels are increased in patients with COPD. (A) Genes upregulated in healthy individuals (*n* = 40) and COPD patients (*n* = 111) from the GSE76925 dataset. (B) Relative KDELR1, KDELR2, and KDELR3 mRNA levels in healthy controls and patients with COPD in the GSE76925 dataset. (C) Haematoxylin and eosin images, AB‐PAS and immunohistochemistry (IHC) analysis of MUC5AC in lung sections from the control group (*n* = 19) and COPD group (*n* = 18). The image parameters were as follows: 1600 × 1200, 72 pixels per inch (PPI). (D) The percentage of goblet cells in the lung field was determined. (E) Quantitative immunohistochemistry (IHC) analysis of MUC5AC in COPD patients (*n* = 18) and control subjects (*n* = 19). (F) Coimmunofluorescence staining of MUC5AC and KDELR2 in the lung tissues of controls and patients with COPD (2048 × 2048, 96 PPI). Scale bars: 50 μm. IOD: Integrated optical density. Data are shown as the mean ± SEM. *p < 0.01 compared to controls. ns, not significant. Two‐tailed unpaired Student's *t*‐tests were used.

To determine whether KDELR is involved in the pathogenesis of COPD, we examined comparative gene expression profiles in the published dataset GSE76925 containing samples from 40 controls and 111 patients with COPD. We focused on ER‐Golgi signalling regulatory characteristics that are responsible for maintaining ER homeostasis. Bioinformatics analysis showed that the expression of KDELR2 in patients with COPD was significantly higher than that in healthy subjects (Figure 1A,B).

AB‐PAS staining was used to semiquantitatively determine airway epithelial mucus abundance. AB‐PAS staining showed that excessive secretions were more abundant in patients with COPD than those in patients without COPD (Figure 1C,D). Consistent with this result, immunohistochemistry showed that MUC5AC expression in COPD patients was much higher than that in control subjects (Figure 1C,E). To explore the location of MUC5AC and KDELR2 in the airway, coimmunofluorescence staining was used to determine the expression of MUC5AC and KDELR2. The colocalization of MUC5AC and KDELR2 was found in the airways of COPD patients (Figure 1F).

### 3.2 KDELR2, but not KDELR3, is overexpressed in the bronchial epithelium of COPD model rats

**FIGURE 2:**
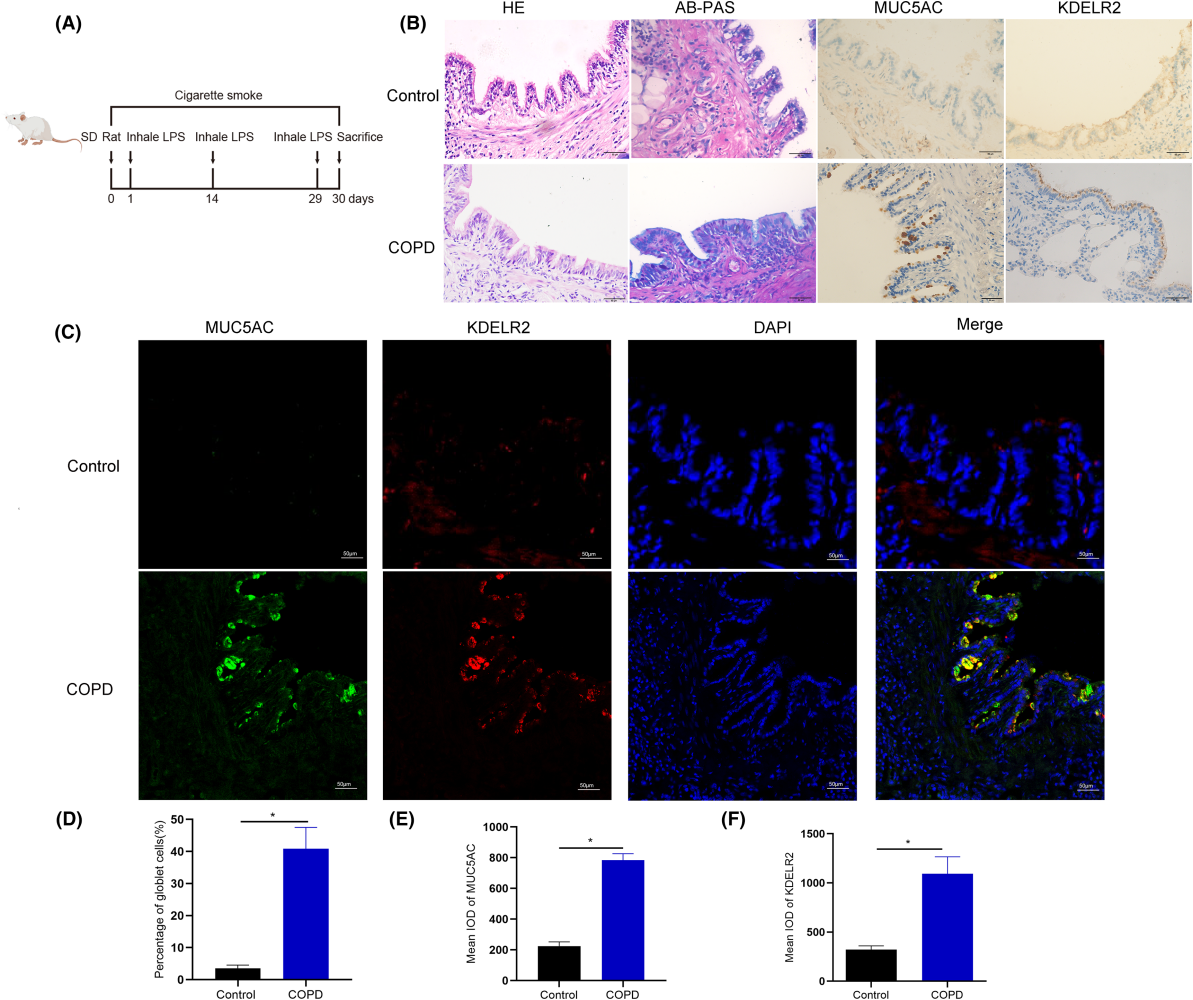
KDELR2 is overexpressed and colocalized with MUC5AC in the bronchial epithelium of rats with COPD. (A) Schematic diagram of the rat model of COPD. (B) Haematoxylin and eosin images, AB‐PAS images and immunohistochemistry (IHC) images of MUC5AC and KDELR2 in lung sections from control rats and COPD model rats (*n* = 5). The image parameters used were 1600 × 1200 and 72 PPI. (C) Coimmunofluorescence staining of MUC5AC and KDELR2 in lung sections from control rats and COPD model rats (*n* = 5). The imaging parameters used were 2048 × 2048 and 96 PPI. (D) The percentage of goblet cells in the airway was determined. (E, F) Quantitative analyses of immunohistochemical data for MUC5AC and KDELR2 in the airways of control rats and rats with COPD. Scale bars: 50 μm. IOD: Integrated optical density. Data are shown as the mean ± SEM. *p < 0.01 compared to the control group. The *p*‐value was obtained by two‐tailed Student's *t‐*test.

To explore the differential expression of KDELR2 and KDELR3 in COPD and normal rat airway epithelium, we examined KDELR2 and KDELR3 expression by immunofluorescence staining and immunohistochemistry. Consistent with the findings in human lung specimens, AB‐PAS and immunohistochemical staining verified the presence of airway mucin hypersecretion in COPD model rats (Figure 2B,D,E). As shown in Figure 2, MUC5AC and KDELR2 staining intensity was significantly greater in the bronchial epithelium of rats with COPD than that of control rats. Furthermore, we found that MUC5AC and KDELR2 were colocalized in the airway (Figure 2C). However, we did not observe any differences in KDELR3 expression between the control group and the COPD group (Figure S2).

### 3.3 UPR mediators involved in ISR are increased in COPD model rats

**FIGURE 3:**
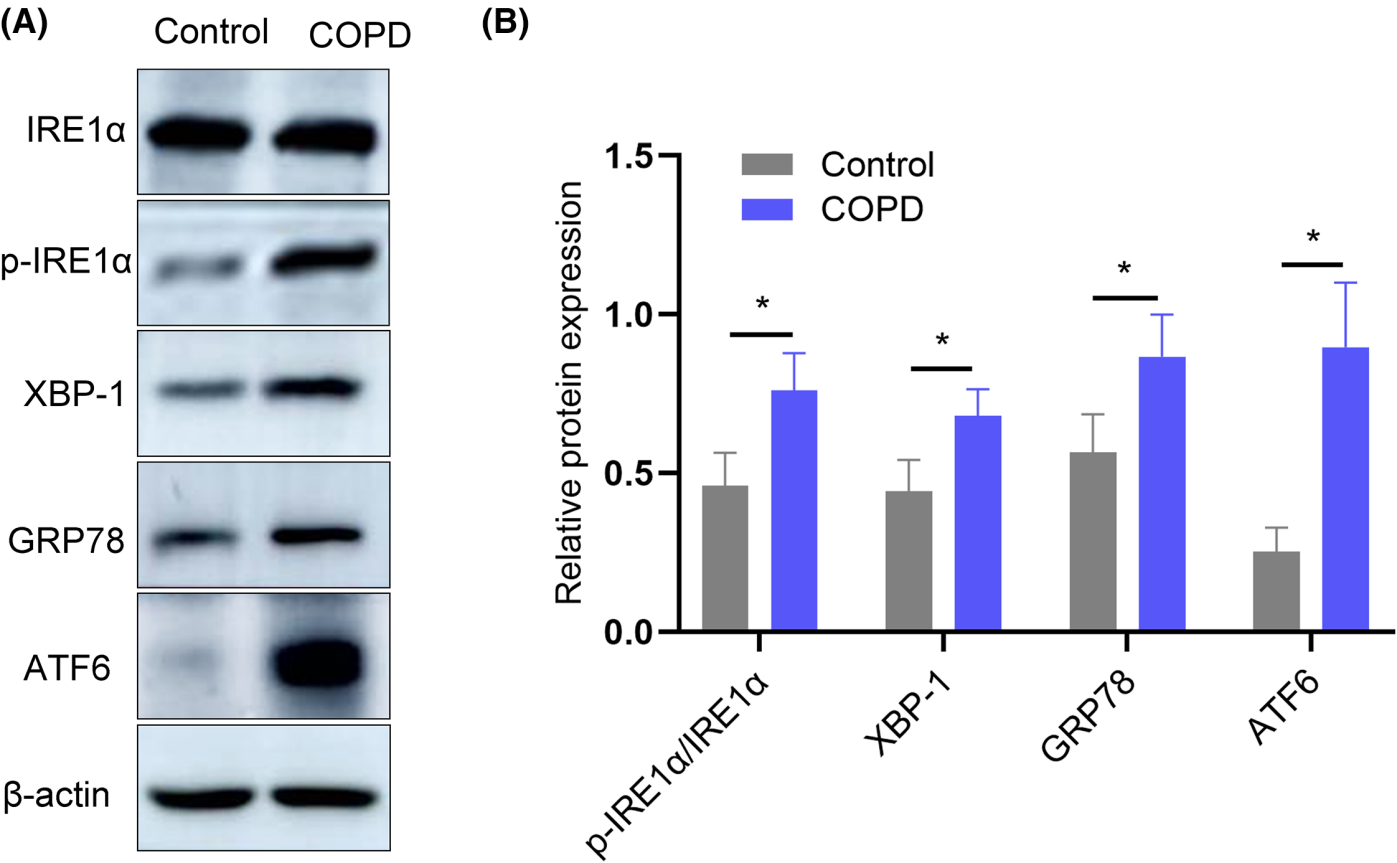
UPR mediator levels are increased in COPD model rats. (A) Representative Western blots of IRE1α, p‐IRE1α, XBP‐1, GRP78 and ATF6 in lung tissues from control rats and COPD model rats. (B) Relative protein expression of p‐IRE1α, XBP‐1, GRP78 and ATF6 measured by Western blotting (*n* = 6). Data are shown as the mean ± SEM. *p < 0.05 compared with the control group. The *p*‐value was calculated by two‐tailed Student's *t‐*test.

It has been shown that the UPR is involved in the regulation of mucus expression.^14^ To explore whether the integrated stress response of the ER occurs in parallel with MUC5AC hypersecretion in rats with COPD, expression levels of typical UPR proteins, such as p‐IRE1α, XBP‐1, GRP78 and ATF6, were determined by Western blotting in lung tissues. Western blot analysis showed that expression of p‐IRE1α, XBP‐1, GRP78 and ATF6 was significantly elevated in the COPD group of rats compared to the control group (all *p* < 0.05, Figure 3A,B).

### 3.4 KDELR2 is responsible for MUC5AC hypersecretion in COPD model rats

**FIGURE 4:**
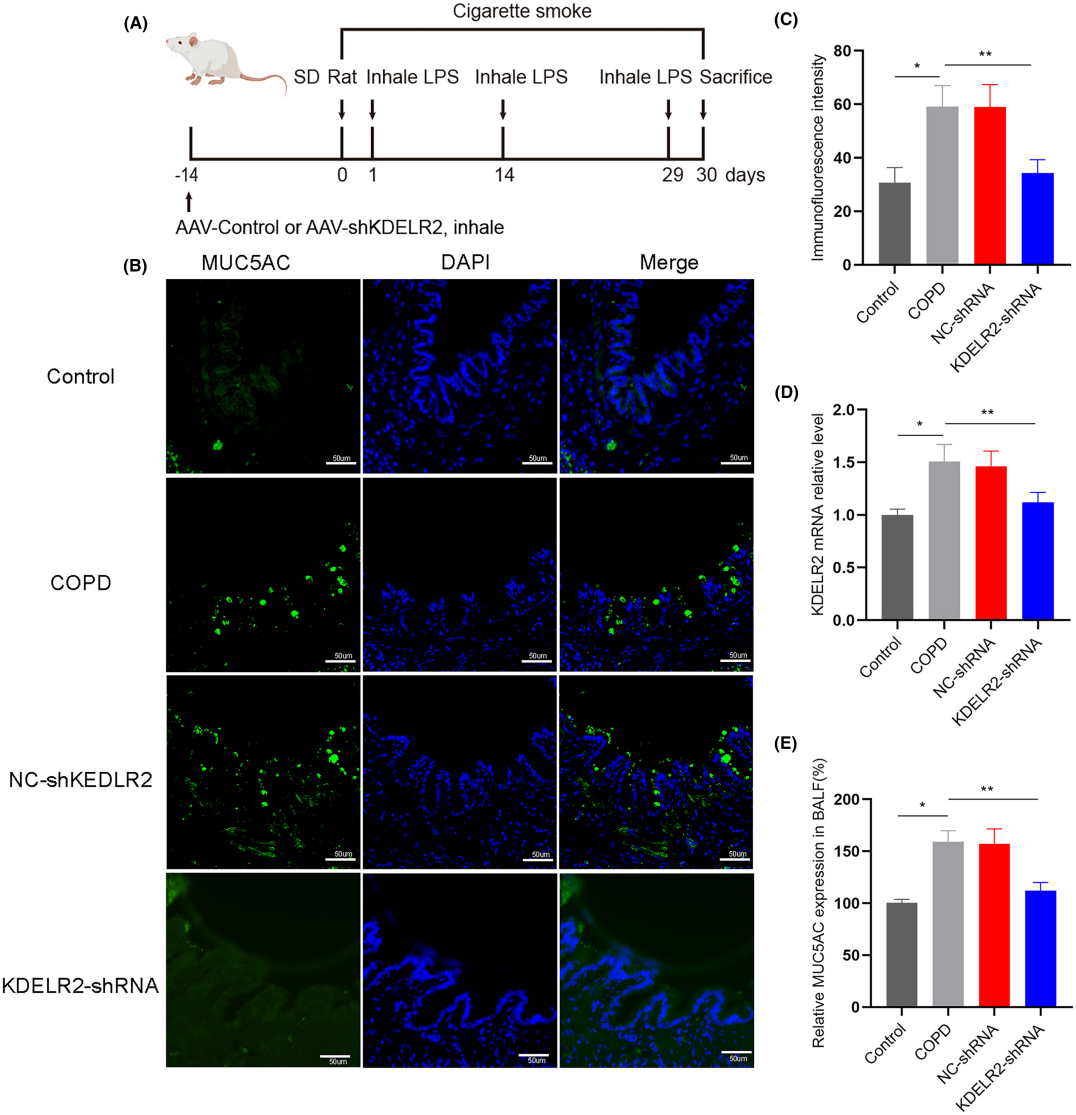
Knockdown of KDELR2 decreased the expression of MUC5AC. (A) Schematic diagram showing the protocol for the administration of AAV containing KDELR2 shRNA or NC shRNA to COPD rats. (B, C) Confocal laser immunofluorescence staining of MUC5AC and quantitative analysis of control rats, COPD model rats, and COPD model rats transfected with AAV negative control shRNA and KDELR2 shRNA (*n* = 6). The imaging parameters were as follows: 2048 × 2048, 96 PPI. Scale bars: 50 μm. (D) Relative mRNA expression of KDELR2 determined by RT–qPCR (*n* = 6). (E) The relative expression of MUC5AC in BALF was determined using ELISA (*n* = 6). Data are shown as the mean ± SEM. *p < 0.05 compared with the control group; **p < 0.05 compared with the COPD group. One‐way ANOVA with Tukey–Kramer post hoc test.

As we found that KDELR2 and MUC5AC expression were increased in parallel in COPD model rats, we next constructed a KDELR2‐knockdown rat model to determine whether KDELR2 plays an essential role in MUC5AC hypersecretion. Rats in the COPD group were transfected with AAV containing KDELR2 shRNA or NC shRNA (Figure 4A). The expression of KDELR2 in rat lung tissues was significantly decreased by KDELR2 shRNA transfection (Figure 4D; Figure S3). Using ELISA and immunofluorescence staining, it was seen that downregulation of KDELR2 caused a reduction in MUC5AC levels in both bronchial epithelium and BALF (Figure 4B,C,E), which supported the hypothesis that KDELR2 might participate in MUC5AC hypersecretion in COPD.

### 3.5 The IRE1α/XBP‐1 s pathway influences KDELR2 expression during MUC5AC overproduction in vivo

**FIGURE 5:**
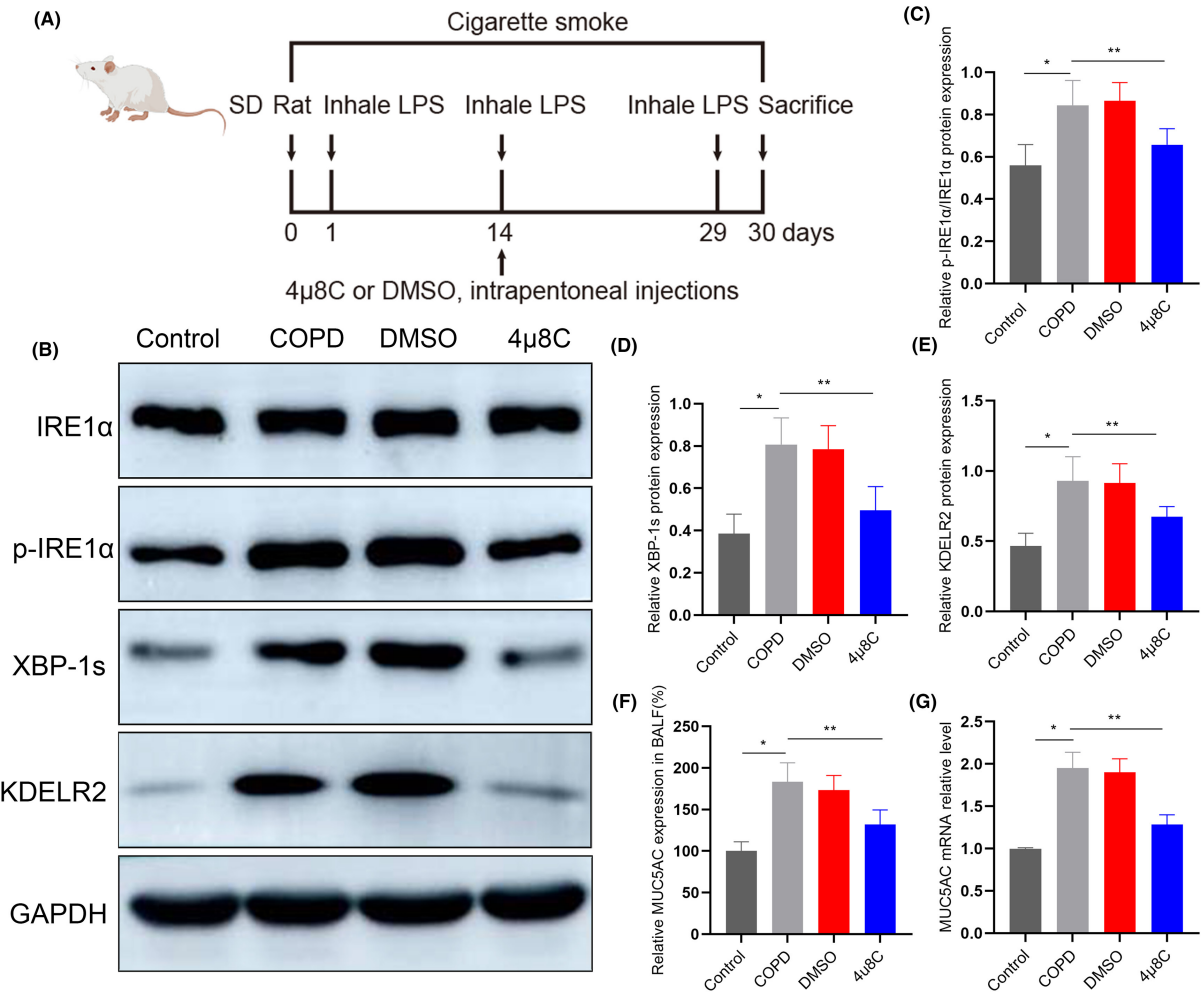
The IRE1α/XBP‐1 s pathway influences KDELR2 expression during MUC5AC overproduction in the airway. (A) Schematic diagram of pharmacological inhibition in rats. (B) The expression of IRE1α, p‐IRE1α, XBP‐1 s and KDELR2 in the lungs of the control group, COPD group, COPD group treated with DMSO and p‐IRE1α inhibitor 4μ8C determined by Western blotting (*n* = 6). (C–E) Relative protein expression of p‐IRE1α/IRE1α, XBP‐1 s and KDELR2 measured by Western blotting (*n* = 6). (F) The relative expression of MUC5AC in BALF was determined by ELISA (*n* = 6). (G) Relative mRNA expression of MUC5AC in lung tissues measured by RT–qPCR (*n* = 6). Data are shown as the mean ± SEM; *p < 0.05 compared with the control group; **p < 0.05 compared with the COPD group. One‐way ANOVA with Tukey–Kramer post hoc test.

To further explore the potential role of IRE1α/XBP‐1s signalling in KDELR2 overexpression in rats with COPD, we next investigated the influence of IRE1α/XBP‐1s signalling on KDELR2 via pharmacological disruption of the IRE1α/XBP‐1s signalling cascade (Figure 5A). After the administration of 4μ8C, a specific IRE1α phosphorylation inhibitor, we analysed the levels of IRE1α, p‐IREα, XBP‐1s and KDELR2 in lung specimens. Western blot analyses showed that the expression of p‐IREα, XBP‐1s and KDELR2 was significantly increased in the COPD model rats. However, these increases were partially attenuated by the administration of 4μ8C in rats with COPD (Figure 5B–E). In addition, we assessed the levels of MUC5AC present in BALF by ELISA and MUC5AC expression in lung tissues by RT‐qPCR. We found that the increased expression of MUC5AC was partially reversed by 4μ8C (Figure 5F,G). At the same time, as shown in Figure 5, administration of 4μ8C also decreased the expression of KDELR2 by Western blot. These results additionally suggest that IRE1α/XBP‐1s signalling plays a crucial role in the overexpression of KDELR2 and MUC5AC.

### 3.6 CCK‐8 assay of cell viability under NE or 4μ8C stimulation

In this in vitro cellular study, we used NE as an acceptable stimulus to mimic the airway epithelial mucus hypersecretion environment in COPD. To determine the proper concentration of NE, a CCK‐8 assay was performed to assess the influence of exposure to NE on cell viability. The results showed that cell viability did not change between 6 and 24 h following treatment with 25–100 ng/mL NE or 2–6 μM 4μ8C. However, exposure to 200 ng/mL NE or 8 μM 4μ8C for 24 h, 100 ng/mL to 200 ng/mL NE or 6 to 8 μM 4μ8C for 48 h significantly reduced cell viability. Thus, 100 ng/mL NE and 6 μM 4μ8C for 24 h were selected as the appropriate concentrations for stimulation (Figures S4 and S9A,D).

### 3.7 NE induces the overexpression of KDELR2 and ISR‐associated proteins, and promotes the ER‐like localization of KDELR2

**FIGURE 6:**
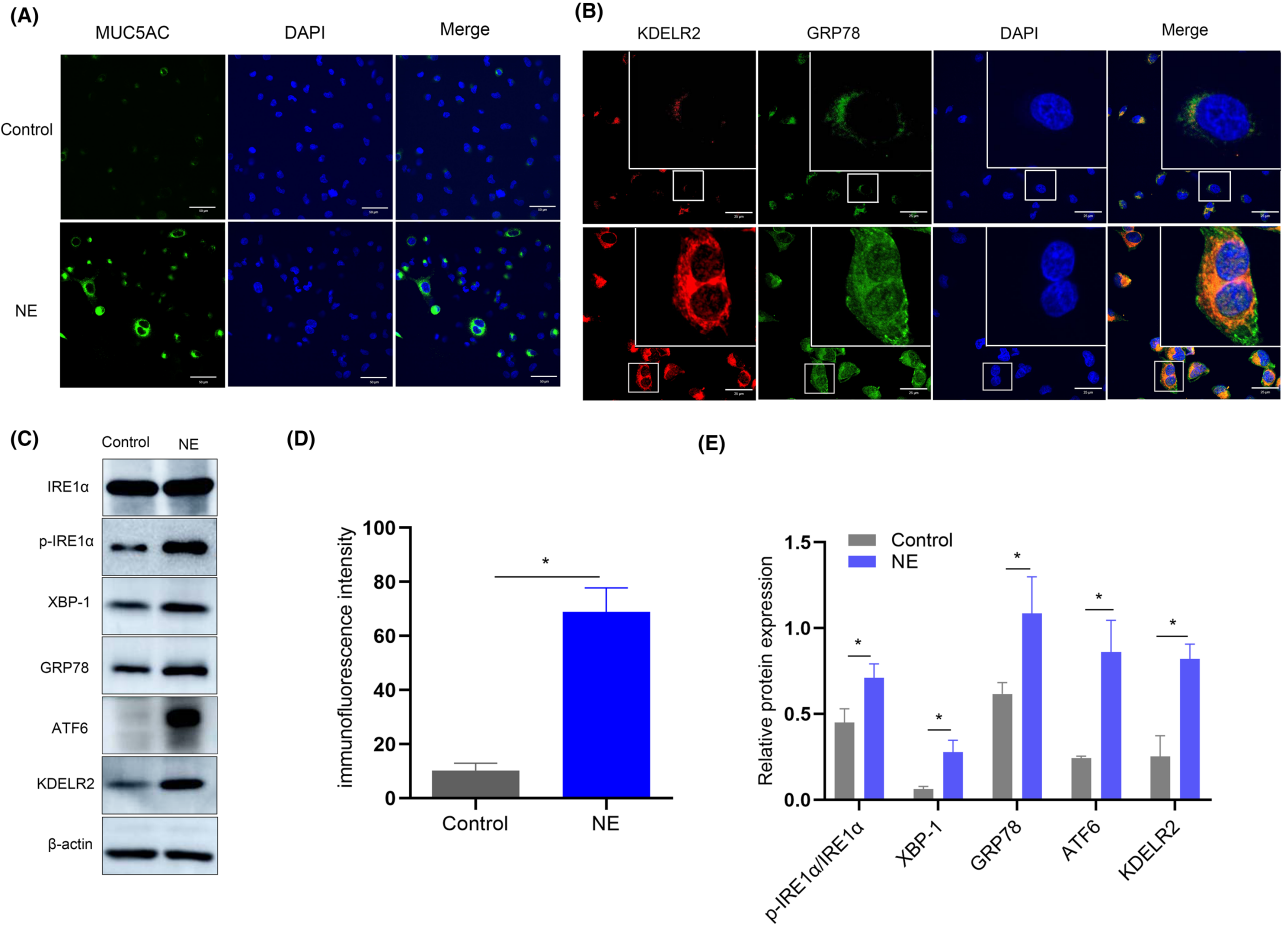
NE induced the expression of MUC5AC, KDELR2, and ISR‐associated proteins and the ER‐like localization of KDELR2 in Calu‐3 cells. (A, D) Confocal laser immunofluorescence staining of MUC5AC and quantitative analysis of control cells and cells treated with NE (*n* = 3). Scale bars: 50 μm. (B) KDELR2 colocalized with GRP78 in close proximity to ER in NE group than the control group as shown by confocal laser immunofluorescence staining (*n* = 3). Pearson's correlation = 0.842. Scale bars: 10 μm. (C, E) Western blot analysis of IRE1α, p‐IRE1α, XBP‐1, GRP78, ATF6 and KDELR2 expression in the control group and NE group (*n* = 3). Data are presented as the means ± SDs. *p < 0.05 compared with the control group. Two‐tailed unpaired Student's *t*‐tests.

To explore the expression of MUC5AC as well as the ISR under NE stimulation, we directly investigated the levels of MUC5AC by immunofluorescence and the levels of KDELR2 and ISR‐associated proteins by Western blotting in both Calu‐3 and BEAS‐2B cells. The intracellular expression levels of MUC5AC, p‐IRE1α, XBP‐1, GRP78, ATF6 and KDELR2 were significantly increased after NE exposure compared with those in untreated Calu‐3 cells (Figure 6A,C–E). A similar profile was observed in BEAS‐2B cells after NE exposure (Figure S5). The colocalization of KDELR2 with GRP78, a core ER chaperone, during exposure to NE was confirmed by means of immunofluorescence (Figure 6B; Figure S5B). NE promoted KDELR2 localization with GRP78 in close proximity to ER in Calu‐3 cells and BEAS‐2B cells.

### 3.8 KDELR2 regulates NE‐induced MUC5AC hypersecretion via ATF6 and IRE1α/XBP‐1s signalling in vitro

**FIGURE 7:**
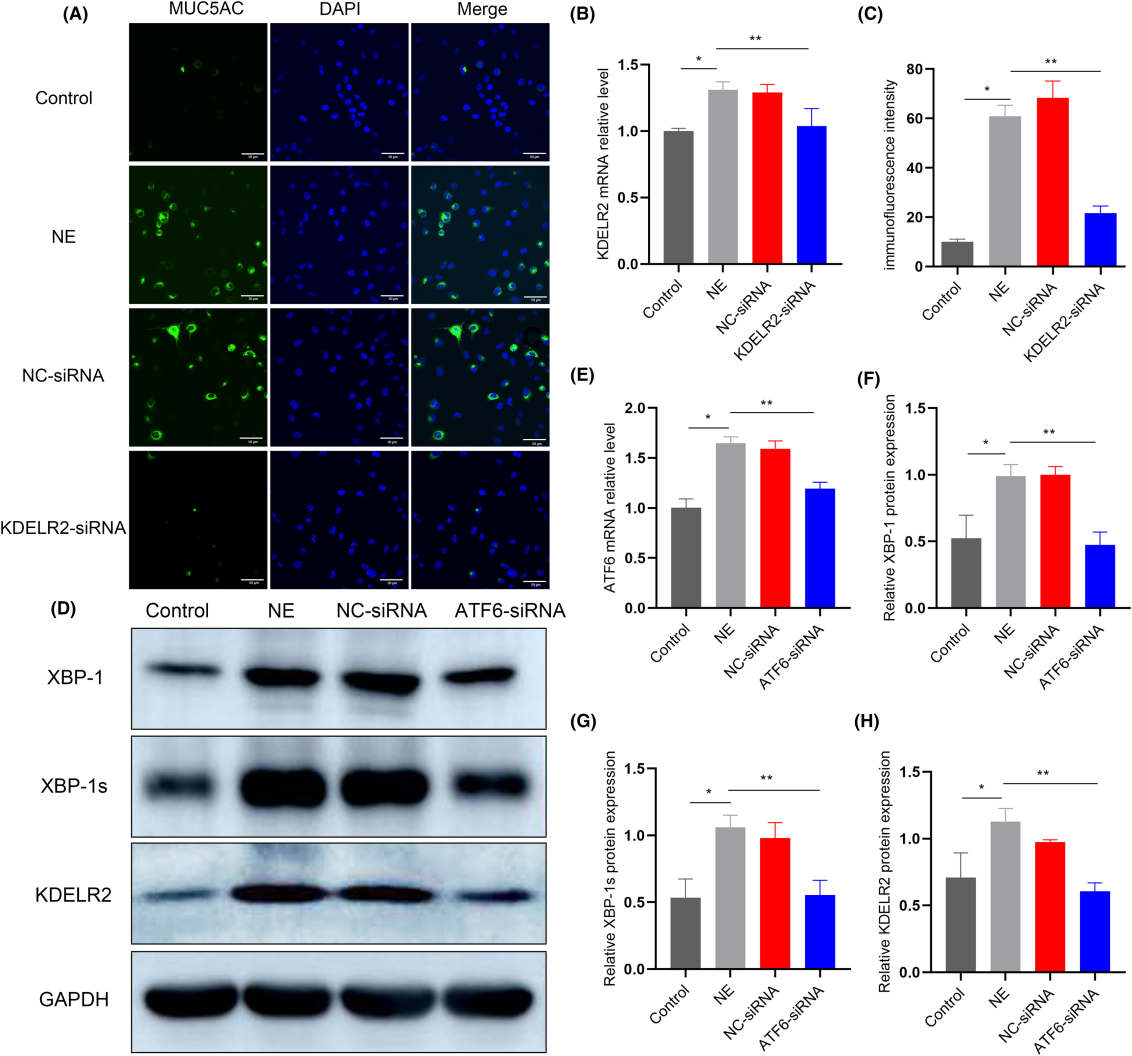
Knockdown of KDELR2 decreased the expression of MUC5AC; and the effect of ATF6 on the expression of KDELR2 induced by NE in Calu‐3 cells. (A, C) Immunofluorescence staining analysis of MUC5AC expression in Calu‐3 cells after NE treatment and siKDELR2 transfection (*n* = 3). Scale bars: 50 μm. (B) Quantitative PCR analysis of KDELR2 gene expression in Calu‐3 cells (*n* = 3). (D, F, G, H) Western blot analysis of XBP‐1, XBP‐1 s and KDELR2 expression after NE exposure and transfection with ATF6 siRNA (*n* = 3). (E) Quantitative PCR analysis of the ATF6 gene after ATF6 siRNA transfection in Calu‐3 cells (*n* = 3). Data are presented as the means ± SDs. *p < 0.01 compared with the control group; **p < 0.01 compared with the NE group. One‐way ANOVA with Tukey–Kramer post hoc test.

KDELRs are located in the ER and are functionally relevant in reducing the intracellular accumulation of unfolded proteins in response to the UPR.^20^ In this study, we confirmed that KDELR2 colocalized with GRP78 on the ER following NE exposure. Thus, we hypothesized that KDELR2 would also be functionally relevant in MUC5AC hypersecretion. We further evaluated the functional role of KDELR2 using siRNA knockdown in Calu‐3 and BEAS‐2B cells (Figure S9B,E). We detected significant downregulation of KDELR2 expression only after transfection with KDELR2 siRNA and confirmed that the KDELR2 siRNA specifically targeted KDEL receptor isoform 2 (Figure 7B; Figures S6B and S7). Importantly, consistent with the in vivo studies, downregulating KDELR2 with a specific siRNA partially attenuated MUC5AC oversynthesis in both Calu‐3 and BEAS‐2B cells under NE stimulation (Figure 7A,C; Figure S6A,C). These results revealed that KDELR2 appears to be functionally relevant for airway MUC5AC overexpression under NE stimulation in vitro.

When the UPR is activated, ATF6, a type‐II ER‐associated transmembrane‐containing transcription factor, is proteolyzed and releases a cytosolic fragment that migrates to the nucleus and binds to the ERS to trigger the transcription of target genes.^21^ To test the role of ATF6 in NE‐mediated KDELR2 induction, we constructed an ATF6 knockdown model using specific siRNA transfection in both Calu‐3 and BEAS‐2B cells (Figure S9C,F). The level of ATF6 decreased after transfection with the ATF6 siRNA (Figure 7E; Figure S6E). Compared with controls, NE induced higher expression levels of XBP1, XBP1s and KDELR2, as measured by Western blot analysis. Upon transfection with ATF6 siRNA, the increased levels of XBP1, XBP1s and KDELR2 in both Calu‐3 and BEAS‐2B cells were partially abolished in response to NE stimulation (Figure 7D,F–H; Figure S6D,F–H).

**FIGURE 8:**
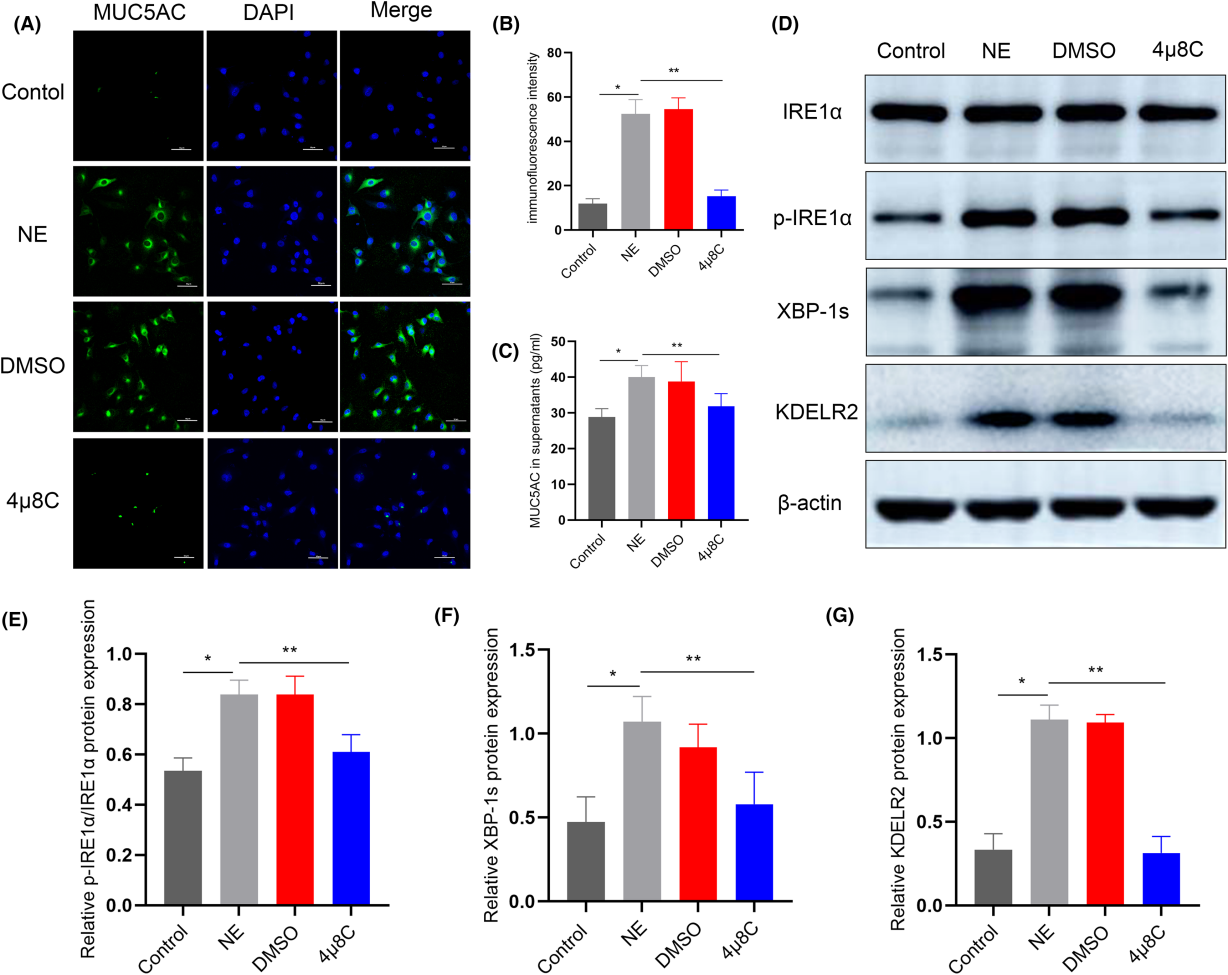
Inhibition of the IRE1α/XBP‐1 s pathway reduced the expression of KDELR2 and MUC5AC in Calu‐3 cells. (A, B) Immunofluorescence staining of MUC5AC and quantitative analysis of Calu‐3 cells treated with NE and the IRE1α inhibitor 4μ8C (*n* = 3). Scale bars: 50 μm. (C) The expression of MUC5AC in culture supernatants was determined by using ELISA (*n* = 6). (D–G) Immunoblotting analysis of IRE1α, p‐IRE1α, XBP‐1 s and KDELR2 in Calu‐3 cells (*n* = 3). Data are presented as the means ± SDs. *p < 0.01 compared with the control group; **p < 0.01 compared with the NE group. One‐way ANOVA with Tukey–Kramer post hoc test.

As studies have suggested that activation of XBP‐1 mRNA splicing plays a crucial role in secretory responses in many cells, including airway epithelia,^22^, ^23^ we evaluated whether NE‐induced KDELR2 expression and MUC5AC overproduction were dependent on IRE1α/XBP‐1 mRNA splicing in vitro. 4μ8C, a specific IRE1α phosphorylation inhibitor, was applied to directly test the functional role of IRE1α/XBP‐1 s signalling in KDELR2 and MUC5AC overexpression in vitro. Compared with control NE‐stimulated cultures, 4μ8C treatments partially attenuated KDELR2 overexpression, accompanied by reduced expression of p‐IRE1α and XBP‐1s (Figure 8D–G; Figure S8D–G). In addition, similar findings were observed in the analysis of intracellular MUC5AC expression and culture supernatants (Figure 8A–C; Figure S8A–C). These results were consistent with the conclusion drawn from rats with COPD subjected to challenge with 4μ8C. These novel findings in vitro suggest that KDELR2 overexpression during MUC5AC hypersecretion is mediated, at least partially, by the activation of ATF6 and IRE1α/XBP‐1s signalling, which in turn is crucial in airway epithelial MUC5AC overproduction.

## 4 DISCUSSION

Airway goblet cell hyperplasia is one of the major pathological features of COPD patients and can lead to mucus hypersecretion and retention in the small bronchus. Thus, continuous MUC5AC hypersecretion in the airways not only contributes to the initiation and progression of COPD but also increases the risk of exacerbation.^3^, ^24^ As major airway secretory cells that generate gel‐forming mucins, airway goblet cells are primarily recognized by ER proliferation.^18^ However, the underlying mechanism by which ER competency in airway goblet cells affects mucus hypersecretion in response to the UPR patients remains to be elucidated. Herein, we provide new insight and focus on the essential role of KDELR2 in airway mucin overproduction. Using a combination of rat models of COPD and cell models, our study demonstrates that the expression levels of MUC5AC are increased in COPD, accompanied by an increased KDELR2 expression level. By knocking down KDELR2, we provide evidence that KDELR2 is a mediator of MUC5AC overexpression, possibly under UPR regulation. Intriguingly, our study indicated that the inhibition of ATF6 and IRE1α/XBP‐1 s could partially block the expression of KDELR2 and MUC5AC.

High levels of airway mucin production in goblet cells are an excessive challenge for both cells and the subcellular organelle ER. A series of specific mechanism are required to cope with protein misfolding and precise modifications in the ER. This demand makes goblet cells specifically vulnerable to ER overloading. Mammalian cells respond to the UPR, resulting in two possible outcomes: (a) reduced protein synthesis, arrested translation, cell apoptosis, or autophagy, or (b) alleviation of protein misfolding and increased expression of ER chaperones to enhance the processing of folded proteins.^25^ Most current studies on the UPR in goblet cells have focused on protein translational arrest, cell apoptosis, or autophagy caused by ER stress, while the adaptive effects of the ER in response to chronic UPR secondary to mild stress have not been fully elucidated.

More research has focused on the role of ER stress in the pathophysiology of COPD in recent years. Numerous studies have shown that ER stress is a major factor in the early development of COPD and is the main trigger of inflammation.^26^, ^27^ A study found that ER stress and UPR‐related protein levels were increased in the lung tissues of rats and mice with COPD exposed to CS.^27^, ^28^ ER stress induced by IL‐13 increases MUC5AC expression in airway epithelial cells, and an inhibitor of ER stress partially attenuates MUC5AC expression in asthma.^29^ Our study confirmed the presence of a UPR response in the lung tissues of COPD model rats with mucus hypersecretion by detecting significantly overexpressed UPR‐related proteins, such as IRE1, ATF6, and XBP1, in lung specimens. Our findings are consistent with several studies using surgical specimens from COPD patients and with the animal investigation mentioned above.^26^, ^30^

ER‐Golgi transport is crucial to protein generation and secretion. KDELR was demonstrated to be critical for the retrieval of ER‐resident molecular chaperones, including a KDEL sequence from the ER‐Golgi intermediate compartment (ERGIC) complex when chaperones are released and escape from the ER.^31^, ^32^ As major ER retrieval mediators, KDELRs contribute to the maintenance of ER homeostasis and quality control of the ER.^33^ There are three KDEL receptors, KDELR1, KDELR2, and KDELR3, encoded by the mammalian genome. Recently, it was discovered that the expression levels of KDELR1, KDELR2 and KDELR3 mRNA increased after exposure to a chemical inducer of the UPR, tunicamycin.^34^ KDELRs are also reported to be involved in autophagy, one of the important mechanisms strictly related to the UPR and responsible for the clearance of misfolded cargoes.^35^ In response to different cellular stimuli, adaptation to the UPR may be realized by different subtypes of KDELRs. Further studies on the functions of different subtypes of KDELRs have shown that the activation of KDELR1, but apparently not KDELR2 or KDELR3, modulates the turnover of lipid droplets via autophagy and relocates lysosomes to sustain secretory processes.^35^ A previous investigation indicated that cells primarily upregulate KDELR2 and KDELR3 to address the increased burden of misfolded proteins and counteract the loss of ER chaperones instead of KDELR1 upregulation.^36^ In our study, we first searched the GEO database using bioinformatics analysis and found significantly increased expression of KDELR2 in the lung specimens of individuals with COPD. Furthermore, we found that the levels of ISR‐associated molecules and KDELR2 are increased in models of mucus hypersecretion both in vivo and in vitro. Thus, in this study, we provide insights into the essential role of KDELR2 in airway mucin hypersecretion. Our in vivo study demonstrated that the overexpression of MUC5AC in COPD models was accompanied by an increased KDELR2 level but not KDELR3 level. Knockdown of KDELR2 partially suppressed the increased MUC5AC levels in COPD model rats, which supports the involvement of KDELR2 in MUC5AC hypersecretion in COPD. In our in vitro study, with immunofluorescence confocal microscopy, KDELR2 was found to localize to ER‐like structures under NE simulation. The results from our investigation support the notion that KDELR2 might play an important role in ER proliferation in airway goblet cells in response to the UPR. ER proliferation subsequently provides a prerequisite for the formation of a certain cellular physiological status that is suitable for goblet cells to accomplish MUC5AC hypersecretion.

In our further study, we demonstrated that the UPR mediators ATF6 and IRE1α/XBP‐1 s might induce KDELR2 signalling during MUC5AC hypersecretion. ATF6, a sensor protein on the ER membrane, acts as both a transcription factor and a UPR transducer.^37^ ATF6 is transferred to the Golgi apparatus when misfolded proteins accumulate in the ER, where it is processed to form a cytosolic fragment that serves as the main mediator of the adaptive response to ER protein misfolding.^38^ The main function of ATF6 is to restore homeostasis. A study found that ATF6 was important in the IRE1‐dependent induction of UPR transcription, which induces gene expression through the regulation of XBP1 activity.^39^ Hiderou et al. confirmed that the transcription factor ATF6 activated the UPR by targeting XBP1.^40^ Interestingly, our in vitro study also showed that ATF6 knockdown downregulated XBP1s and KDELR2 expression. As one of the typical regulators of the UPR, activated IRE‐1α splices 26 nucleotides from the mRNA of the transcription factor XBP‐1 and transforms unspliced XBP‐1 mRNA into an active transcription factor spliced XBP‐1 (XBP‐1s) mRNA triggered by injury and inflammation.^41^ Treatment with 4μ8C, a specific IRE1α phosphorylation inhibitor, decreased the levels of ISR‐associated proteins and KDELR2 and prevented the development of MUC5AC overexpression in vivo and in vitro. Our conclusion regarding the upstream signalling of upregulated KDELR2 under airway MUC5AC hypersecretion is consistent with the present evidence that an approximate threefold increase in KDELR was reported by using Tet‐inducible spliced XBP1 during ER stress, and KDELR genes contain XBP‐1 binding sites.^20^, ^42^ Previous data suggested that KDELR2 and KDELR3 have more XBP‐1 binding sites than does KDELR1, which may indicate a closer correlation with the increased expression of KDELR2 in response to ER stress.^20^ We speculate that the predominant increase in the expression of the KDELR2 subtype in the COPD lung specimens in our study, likely confirms the same mechanism revealed in previous research. However, additional studies may be needed in the future to confirm this hypothesis.

## 5 CONCLUSIONS

In summary, we have revealed a novel molecular mechanism for airway MUC5AC hypersecretion in COPD. We demonstrated that KDELR2 may play a crucial role in this process and may be a prerequisite for MUC5AC hypersecretion in airway goblet cells. KDELR2 upregulation is mainly aimed at adaptive regulation of the UPR and is regulated by upstream ATF6 and IRE1α/XBP‐1s signalling. Our findings might provide rational evidence for developing therapeutic strategies to prevent and treat mucus hyperproduction in COPD patients.

## AUTHOR CONTRIBUTIONS

**Xiaojuan Wu:** Methodology (lead); validation (equal); writing – original draft (lead). **Fawang Du:** Investigation (equal); methodology (equal); software (equal). **Aijie Zhang:** Methodology (equal); resources (equal). **Guoyue Zhang:** Data curation (equal). **Rui Xu:** Conceptualization (equal); methodology (equal); writing – review and editing (equal). **Xianzhi Du:** Supervision (equal); writing – review and editing (equal).

## FUNDING INFORMATION

This study was supported by grants from Chongqing Natural Science Foundation (Grant No cstc2021jcyj‐msxmX0216); Chongqing medical scientific research project (Joint project of Chongqing Health Commission and Science and Technology Bureau, Grant No 2022MSXM144); Program for Youth Innovation in Future Medicine, Chongqing Medical University (Grant No W0118); The First batch of key Disciplines On Public Health in Chongqing (Grant No CMHC: (2022) No. 71).

## CONFLICT OF INTEREST STATEMENT

The authors confirm that there are no conflicts of interest.

## Supporting information


Figure S1.

Figure S2.

Figure S3.

Figure S4.

Figure S5.

Figure S6.

Figure S7.

Figure S8.

Figure S9.

## Data Availability

The data during this study are available from the corresponding author on a reasonable.
