# Supplementary figures and images for: KDELR2 is necessary for chronic obstructive pulmonary disease airway Mucin5AC hypersecretion via an IRE1α/XBP‐1s‐dependent mechanism
Source: J Cell Mol Med. 2024 Oct 4;28(19):e70125. doi: 10.1111/jcmm.70125 (PMC11451269; doi:10.1111/jcmm.70125)

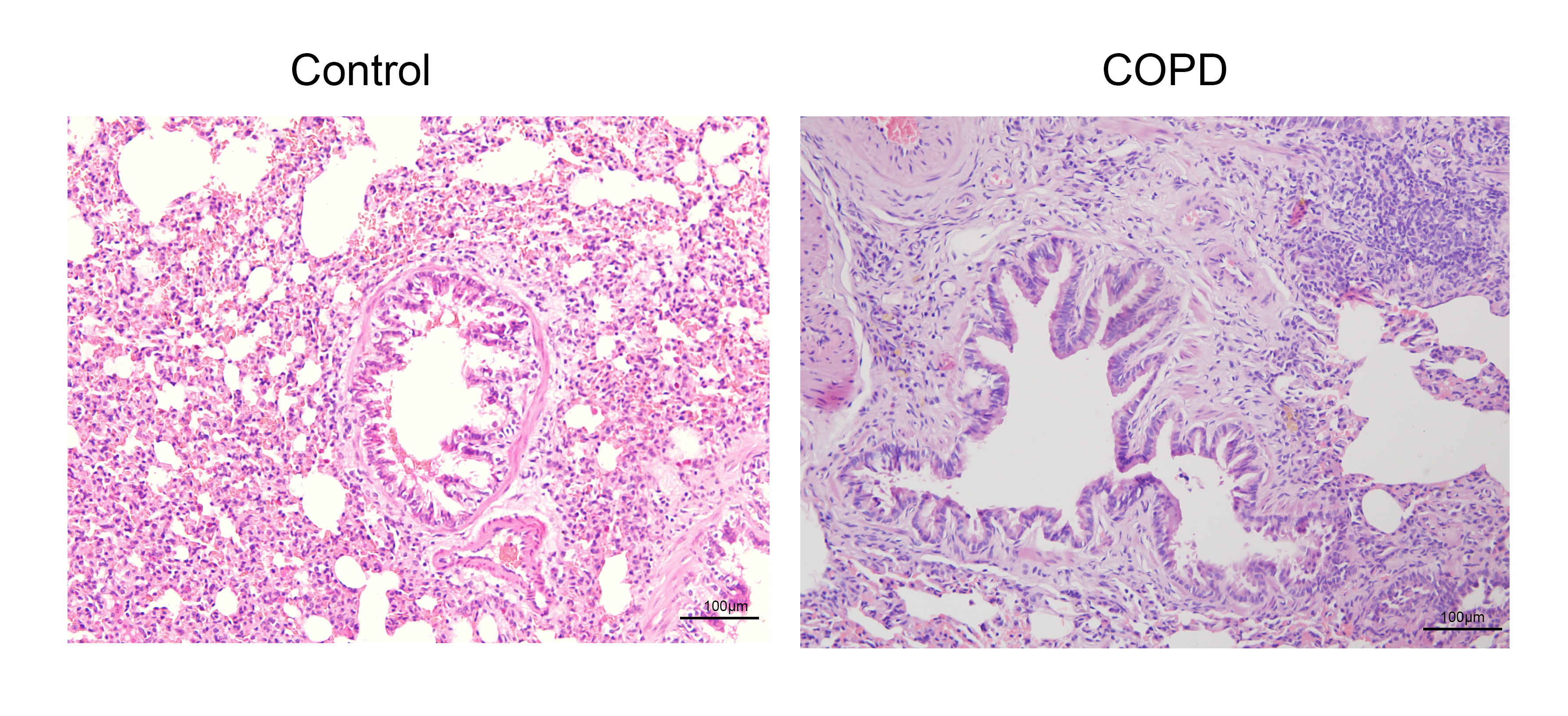

Supplement: Supplementary file 1 — Figure S1. [file JCMM-28-e70125-s002.tif]

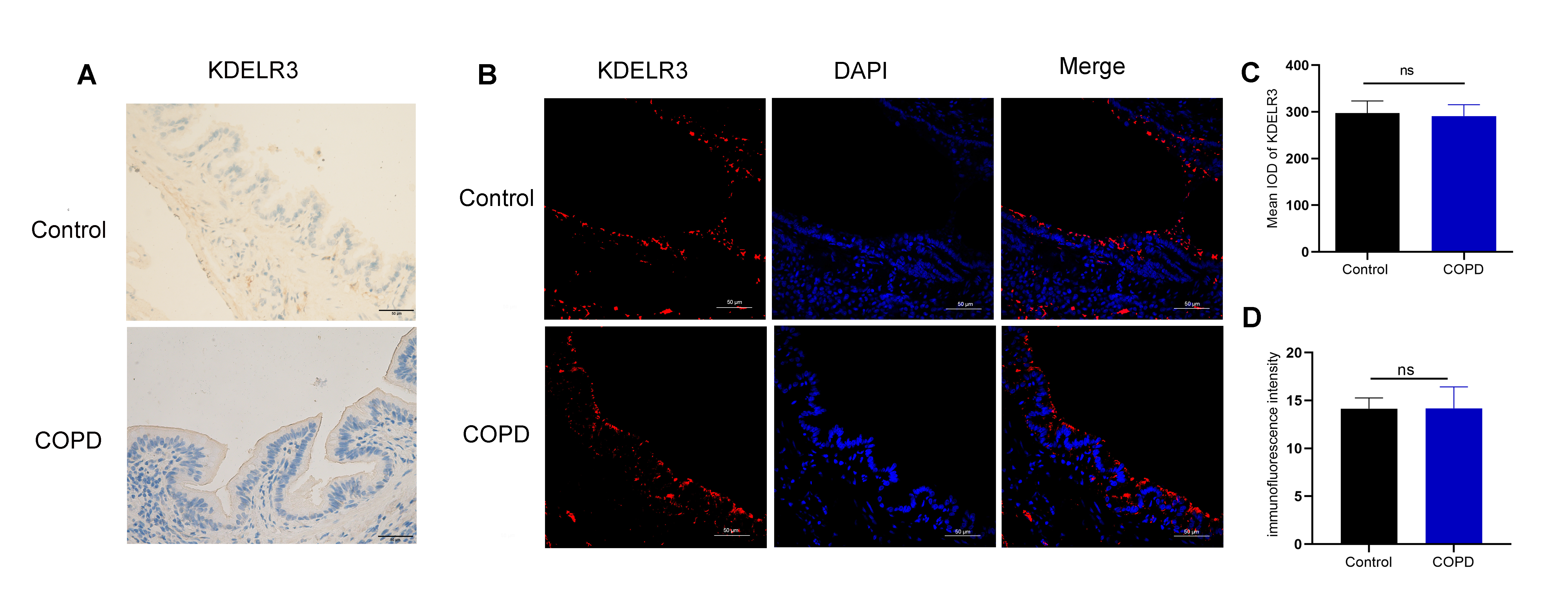

Supplement: Supplementary file 2 — Figure S2. [file JCMM-28-e70125-s003.tif]

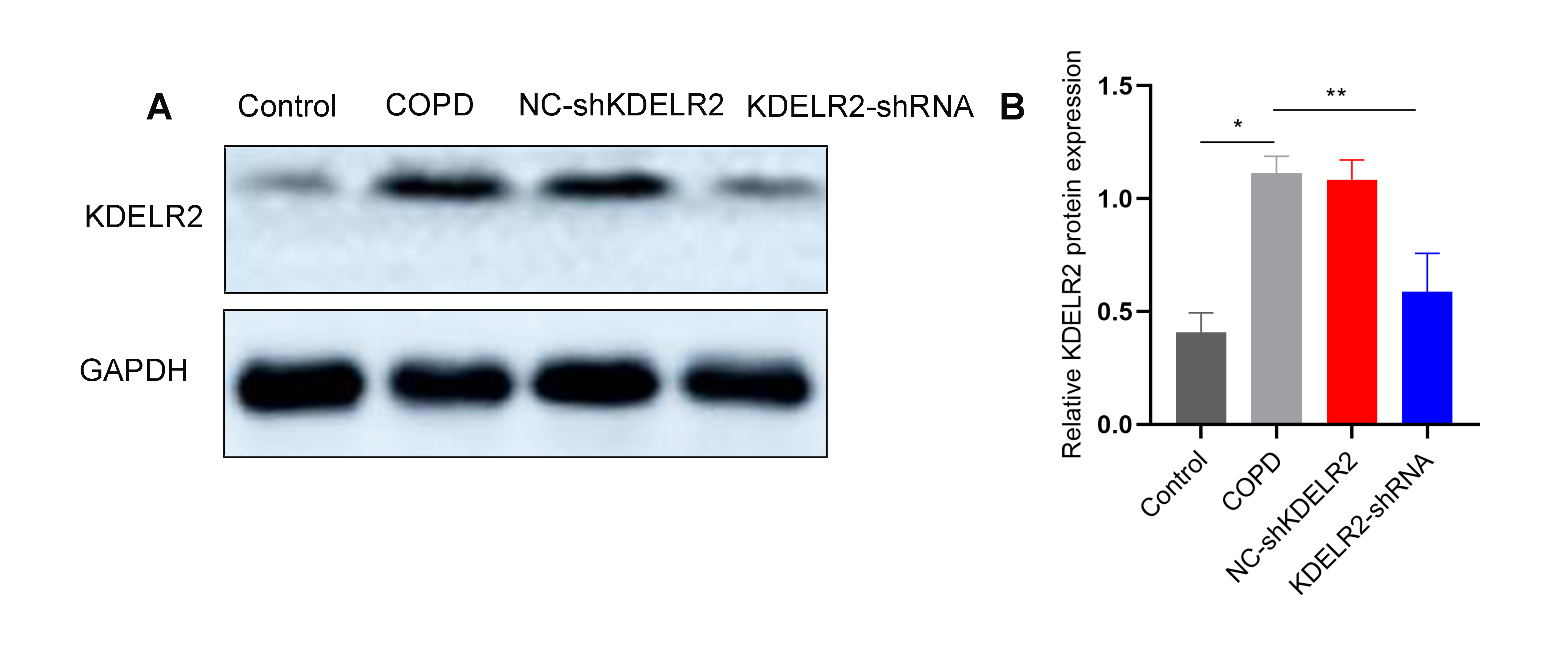

Supplement: Supplementary file 3 — Figure S3. [file JCMM-28-e70125-s007.tif]

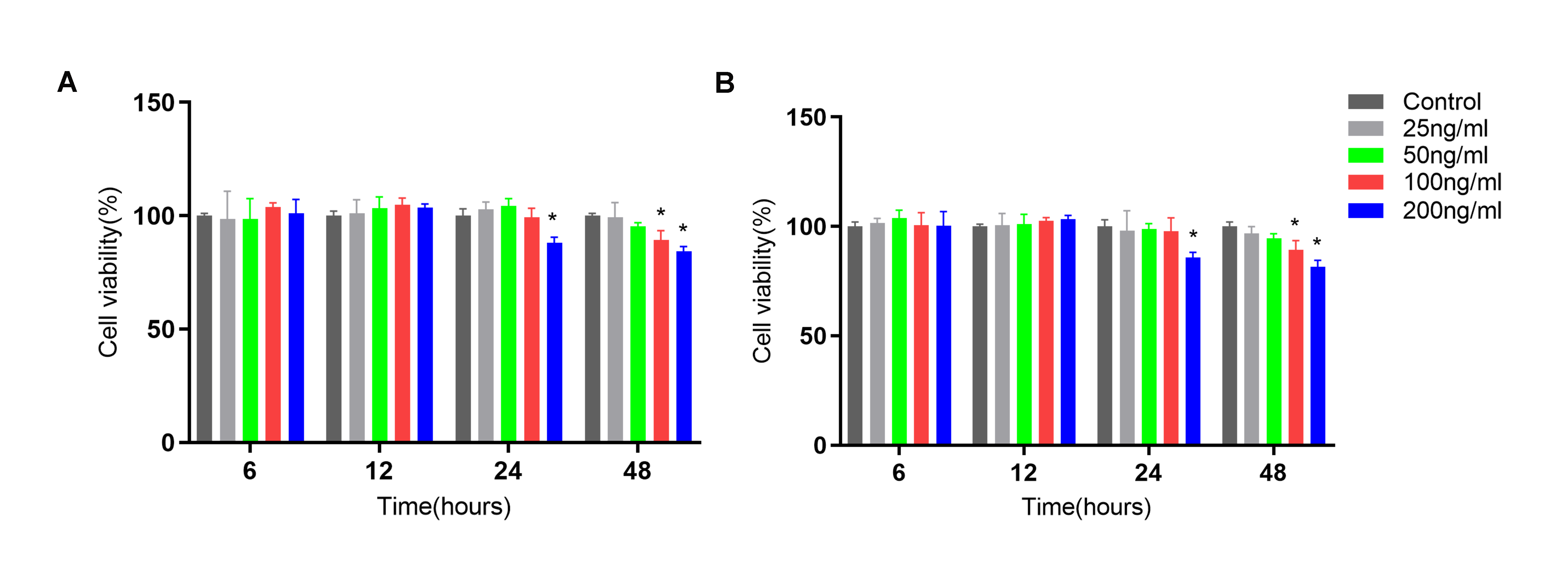

Supplement: Supplementary file 4 — Figure S4. [file JCMM-28-e70125-s009.tif]

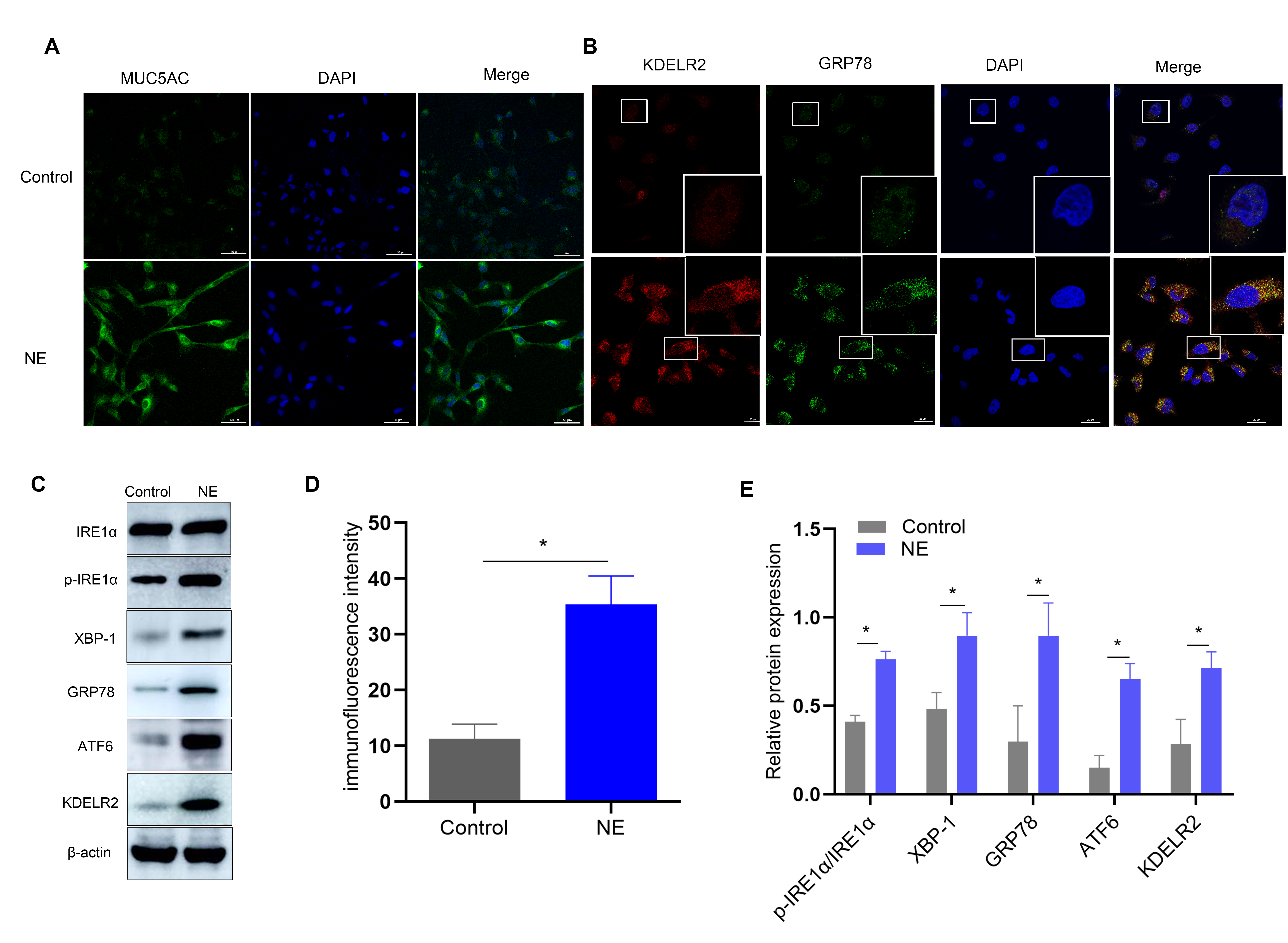

Supplement: Supplementary file 5 — Figure S5. [file JCMM-28-e70125-s008.tif]

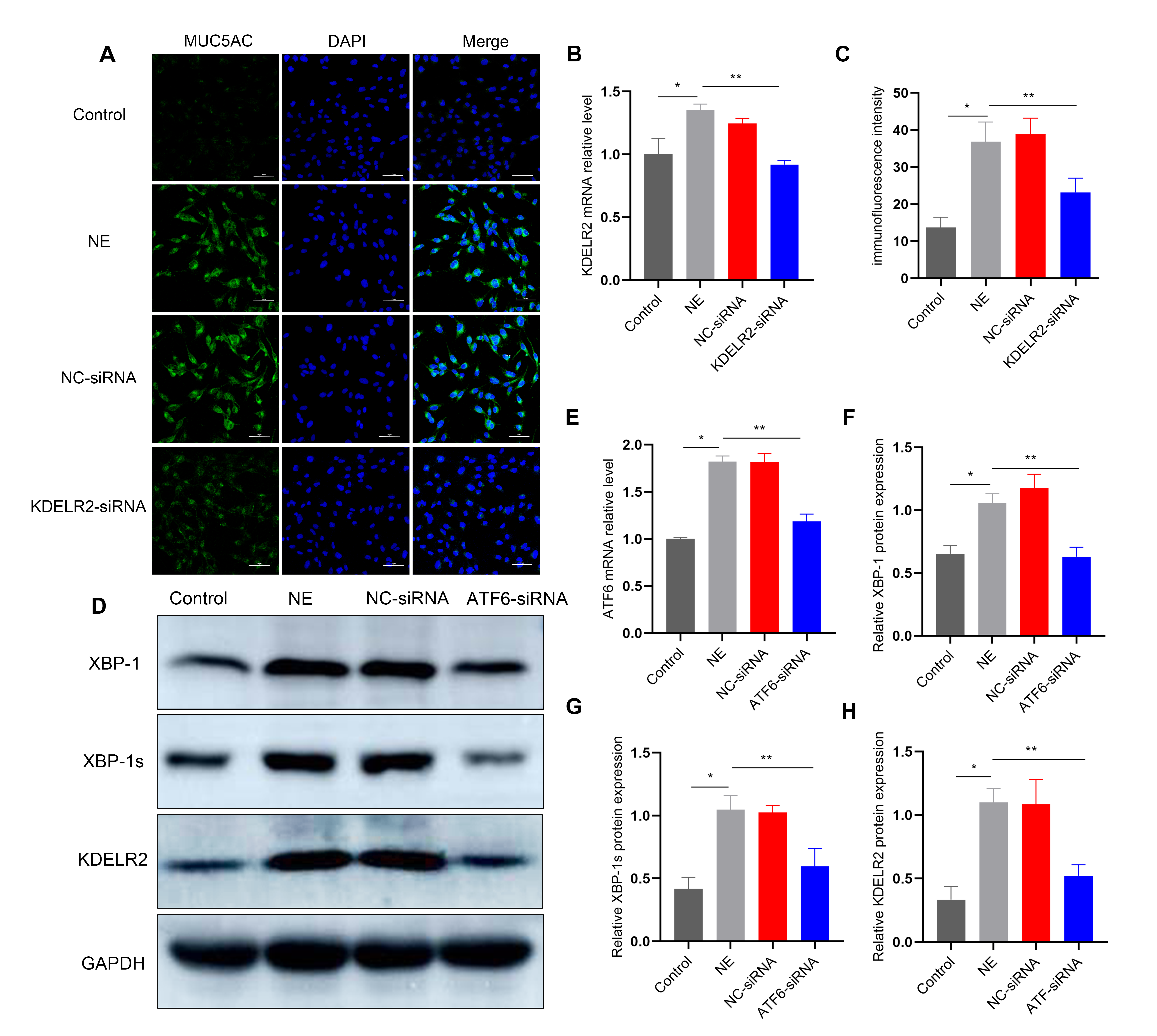

Supplement: Supplementary file 6 — Figure S6. [file JCMM-28-e70125-s004.tif]

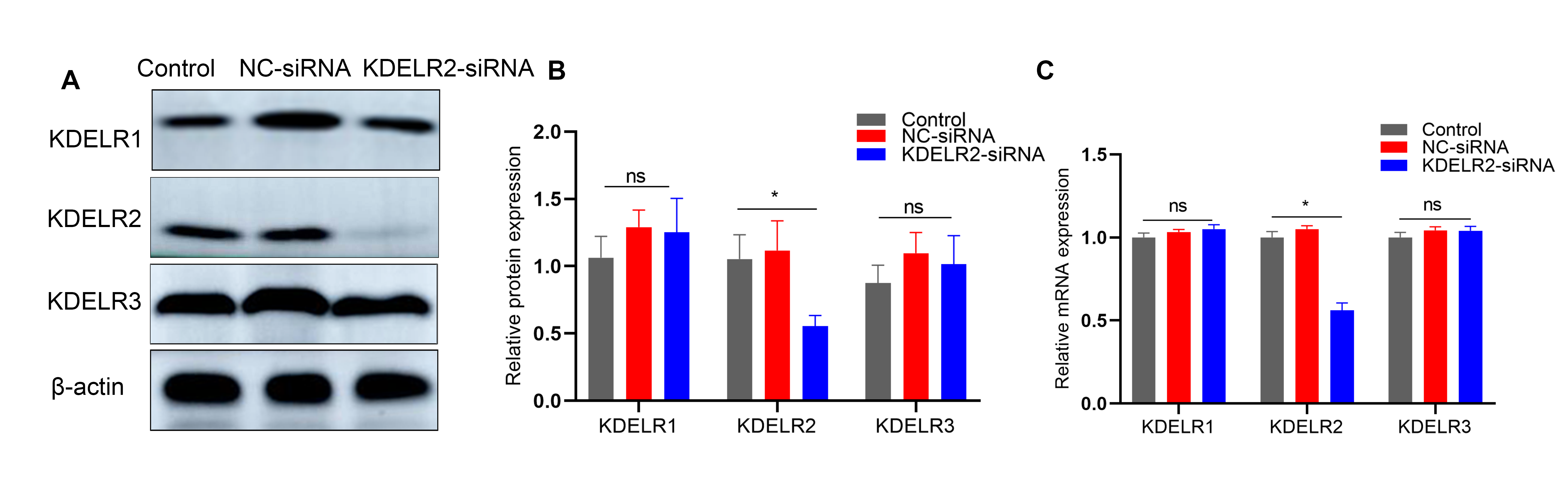

Supplement: Supplementary file 7 — Figure S7. [file JCMM-28-e70125-s005.tif]

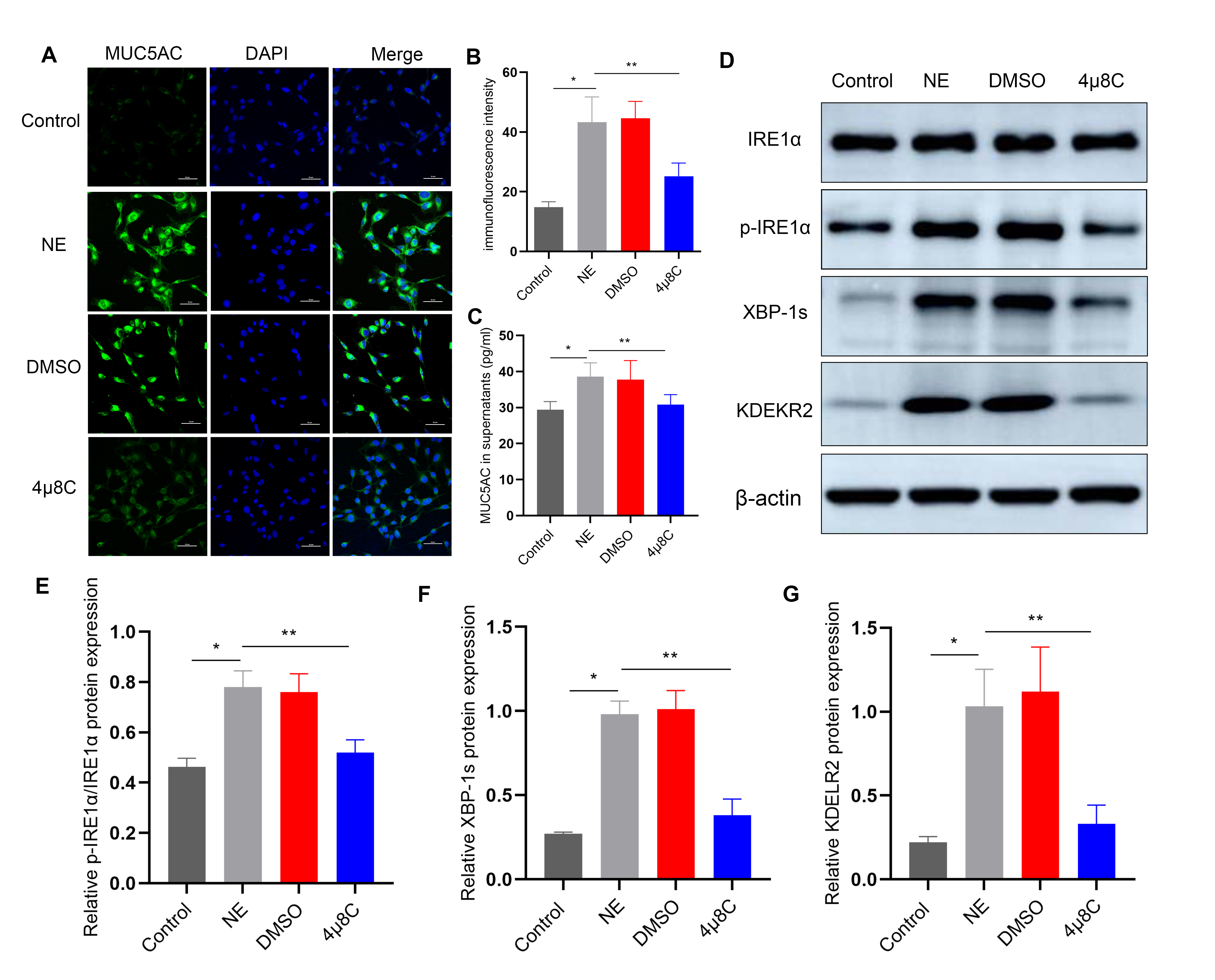

Supplement: Supplementary file 8 — Figure S8. [file JCMM-28-e70125-s006.tif]

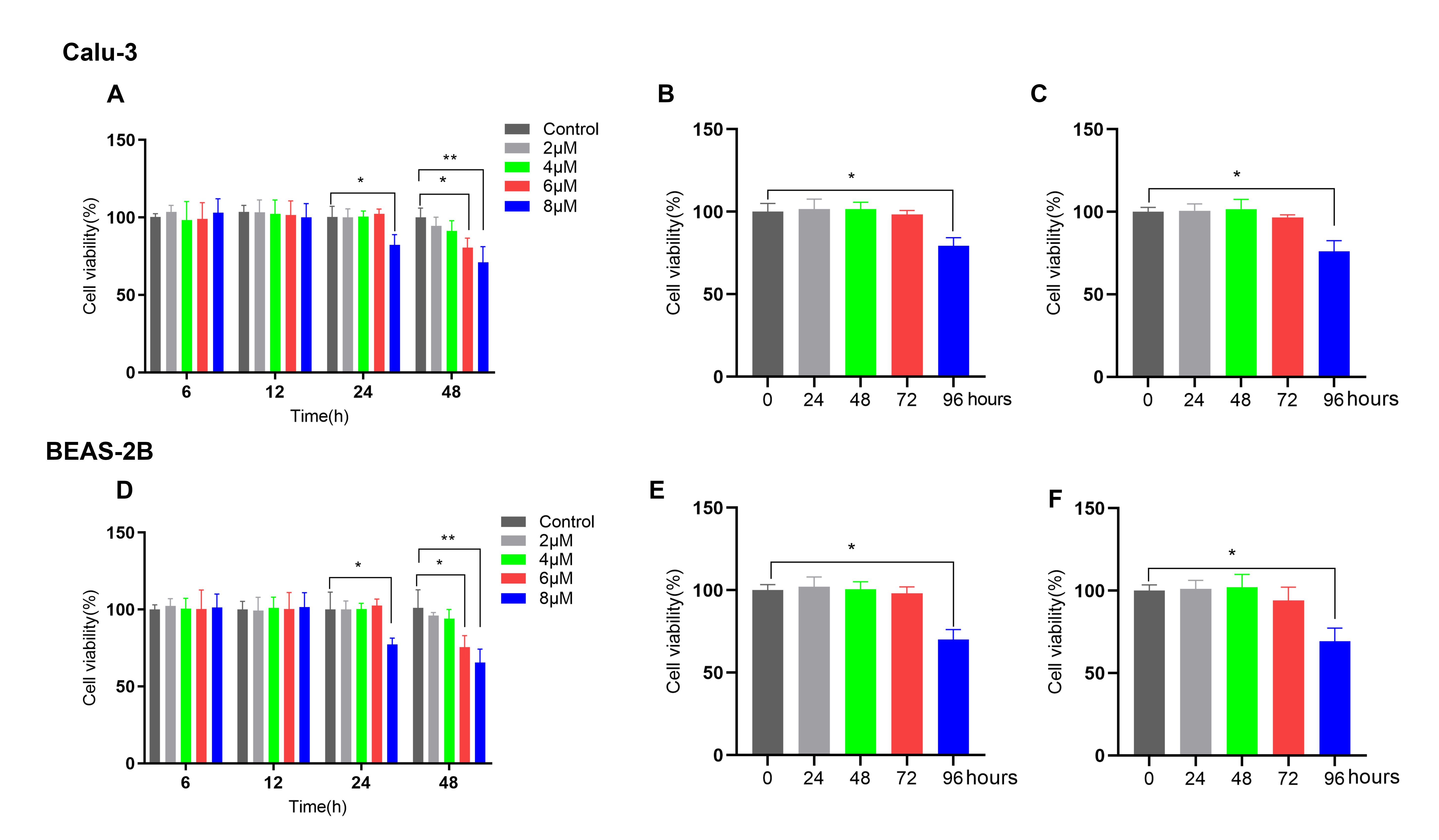

Supplement: Supplementary file 9 — Figure S9. [file JCMM-28-e70125-s001.tif]
